# Rare Genetic Diseases: Nature's Experiments on Human Development

**DOI:** 10.1016/j.isci.2020.101123

**Published:** 2020-05-01

**Authors:** Chelsea E. Lee, Kaela S. Singleton, Melissa Wallin, Victor Faundez

**Affiliations:** 1 Department of Cell Biology, Emory University, Atlanta, GA 30322, USA

**Keywords:** Clinical Genetics, Human Genetics

## Abstract

Rare genetic diseases are the result of a continuous forward genetic screen that nature is conducting on humans. Here, we present epistemological and systems biology arguments highlighting the importance of studying these rare genetic diseases. We contend that the expanding catalog of mutations in ∼4,000 genes, which cause ∼6,500 diseases and their annotated phenotypes, offer a wide landscape for discovering fundamental mechanisms required for human development and involved in common diseases. Rare afflictions disproportionately affect the nervous system in children, but paradoxically, the majority of these disease-causing genes are evolutionarily ancient and ubiquitously expressed in human tissues. We propose that the biased prevalence of childhood rare diseases affecting nervous tissue results from the topological complexity of the protein interaction networks formed by ubiquitous and ancient proteins encoded by childhood disease genes. Finally, we illustrate these principles discussing Menkes disease, an example of the discovery power afforded by rare diseases.

## Graphical Abstract

**Figure:**
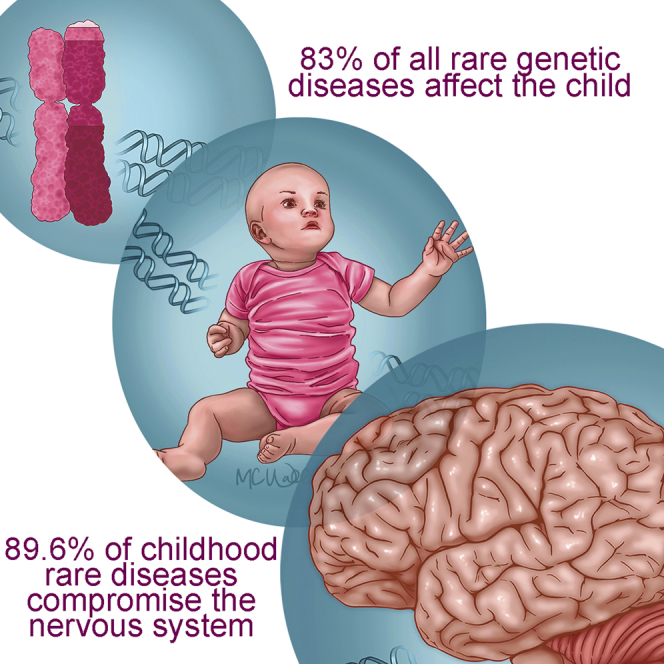

The term “rare disease” describes a group of diseases whose prevalence is so low that they are considered an unviable market for therapeutics in the absence of appropriate incentives and support and too rare to be fully investigated and appropriately managed by professionals (Orphanet, 2020, Waxman, 1983). Among this group, there is a subgroup, the rare genetic diseases target of this article, which frequently affect the nervous system with chronic, progressive, and degenerative pathology that disproportionately affects children (Figure 1). Although the name emphasizes that these diseases are infrequent, the reality is that ∼6,000 rare diseases collectively affect an estimated ∼300 million people worldwide (Nguengang Wakap et al., 2020). In this article, we argue that rare genetic diseases offer a vast landscape for discovery of fundamental and novel biological mechanisms. We will exemplify this concept with three Nobel prizes born out of the study of unique mutations in model genetic organisms. We will discuss examples of how rare monogenic defects have impacted our understanding of important biological problems such as ossification. We will finish this article discussing a rare monogenic disorder of copper metabolism, Menkes disease, because it illustrates how the study of a rare genetic affliction can produce pioneer insight into fundamental biology and prevalent diseases, such as Parkinson disease. The genetic tractability of monogenic defects, their penetrance in early life, and the expanding collection of human mutations and curated phenotypes make rare diseases ideal targets of study for discovering evolutionarily conserved biological mechanisms that go awry in common human diseases. The present and expanding sophistication of genome-wide expression analysis tools, such as genealogical and interactome proteomics (Comstra et al., 2017, Gokhale et al., 2012, Gokhale et al., 2019, Perez-Cornejo et al., 2012, Zlatic et al., 2018), lends itself to comprehensive study of these diseases as a way to deepen our understanding of how a defective protein node participates in the pathogenesis of rare and common diseases.

**Figure 1:**
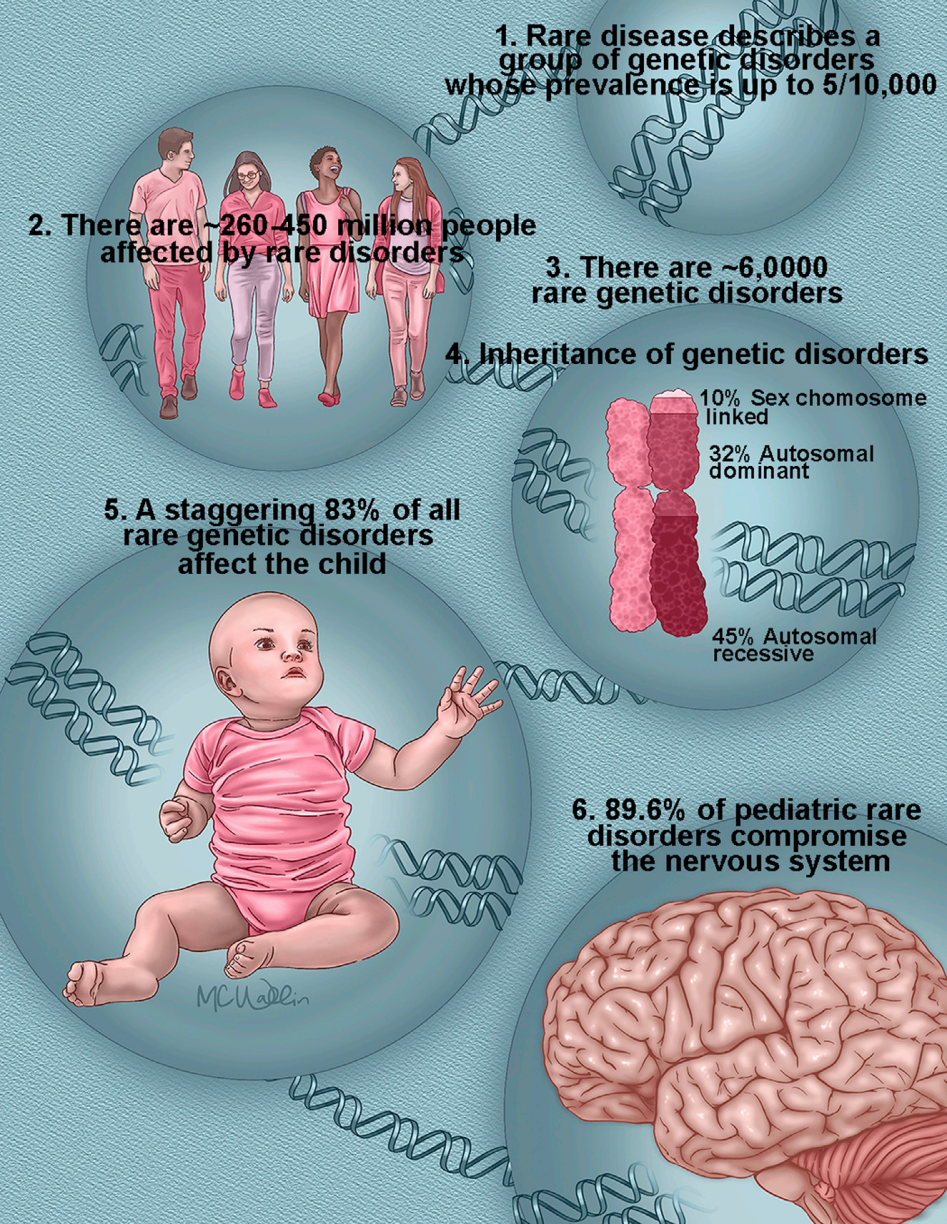
Principal Features of Rare Genetic Disorders

## What Are Rare Diseases?

Rare diseases are defined by their prevalence rather than by unifying pathological or clinical characteristics. In United States, a disease falls in this category if it affects fewer than 200,000 individuals, corresponding to a prevalence of ∼6.6 in 10,000 subjects. This threshold was adopted by law with the passing of the Orphan Product Act of 1983, a law that successfully achieved its goal of accelerating the therapeutic discoveries for treating rare diseases (Boat, 2015, Haffner, 2006, Waxman, 1983). Other countries have defined rare diseases similarly, with thresholds set at a prevalence of 3.9/10,000 in Japan or 5/10,000 in European countries (Boat, 2015). These definitions capture between 6,000 and 10,000 rare diseases that collectively afflict millions of individuals in the United States and an estimated ∼260–450 million people worldwide (Haendel et al., 2020, Nguengang Wakap et al., 2020).

Because rare diseases are defined by their low prevalence, they encompass a broad spectrum of pathologies and pathogenesis mechanisms (Boat, 2015, Orphanet, 2020). Genetic rare diseases are the focus of our article, accounting for nearly 80% of all rare diseases (Boat, 2015, Wright et al., 2018). However, rare diseases also include non-genetic rare diseases such as autoimmune disorders and rare cancers as well as maladies caused by infectious or toxic agents (Orphanet, 2020). Some are known to the general population, such as rabies, but others are exceptionally rare. This is the case of Lemierre syndrome, a septic thrombophlebitis of the head and neck caused by *Fusobacterium necrophorum*, an anaerobic oral commensal (Han, 2015). Arsenic and mercury poisoning can also cause rare diseases, and mesothelioma is a neoplasia caused by asbestos exposure. Finally, there is a miscellaneous category that includes some rare nutritional deficiencies, such as beriberi, and complications of trauma or cancer treatments (Orphanet, 2020).

The unmet medical needs and the plight of affected patients and families are powerful arguments for further study of this wide range of rare diseases, as has been discussed previously (Klein and Gahl, 2018, Schieppati et al., 2008, Wastfelt et al., 2006). Here we will focus on biological arguments as justification for the study of rare diseases. Our argument is that rare genetic diseases are a gateway for discovering novel biology with broad impact for common human diseases. We begin to support this contention analyzing three examples where the study of a rare genetic mutation produced biological insight whose fundamental nature was recognized by Nobel awards. We end this article describing the impact of a rare genetic disorder, Menkes disease, in our knowledge of trace metal biology and the intersection of trace metals with common neurodegenerative diseases.

## Why Study Rare Genetic Diseases, an Epistemological Perspective

There are contrasting views about the value of a rare event in biology and medicine. On one side for a biologist, a rare mutation and its phenotype are windows into mechanisms governing otherwise inscrutable complex biological processes. This vision motivates biologists to study connections between mutation and phenotype. We often perform these studies in model organisms with identical genomes, or isogenic, such as *Saccharomyces cerevisiae*, *Drosophila melanogaster*, or *Mus musculus*. Forward genetic screens seeking mutants in cell secretion, cell cycle, or circadian rhythms are good examples of how just one or few mutants can open doors to progressively unravel the intricacies of these biological processes. For example, the first *cdc* genes required for cell-cycle progression were identified in the Baker's yeast *Saccharomyces cerevisiae* and the fission yeast *Schizosaccharomyces pombe* (Hartwell, 1974, Hartwell et al., 1970, Nurse, 1975). The *sec* genes required for protein secretion were identified in the Baker's yeast (Novick et al., 1980, Novick et al., 1981). The first circadian rhythm gene, *period,* was discovered in the fly (Bargiello et al., 1984, Konopka and Benzer, 1971, Young, 2018). The common thread linking these apparently disparate stories is that a single and rare genetic mutation was sufficient and necessary to begin disentangling these complex biological processes. The knowledge gained in these studies in non-human organisms guided the understanding of disease mechanisms in later discovered rare diseases caused by mutation in human orthologues of these genes (Russo et al., 2013). We document this idea in Table 1, listing the first 23 secretory sec genes and their human orthologues with some of the rare diseases so far identified. These stories of cell cycle, secretion, or circadian rhythms all culminated in Nobel Prizes in 2001, 2013, and 2017, respectively. These stories have spurred considerable research into human diseases ranging from diabetes mellitus to cancer, and these studies span from mechanisms governing insulin secretion by pancreatic beta cells to cell-cycle progression in cancer cells founded on knowledge obtained from these isogenic model genetic organisms (Gerber and Sudhof, 2002, Hartwell and Kastan, 1994). These examples underscore the power of the systematic study of a rare genetic mutation for our understanding of universal biological processes with relevance for prevalent human disease.

**Table 1:** List of the 23 Sec Genes, Human Orthologues, and Paralogues plus Rare Diseases

Yeast Gene	Human Gene	Disease	OMIM #	ORPHANET #	Prevalence
sec1	STXBP1	Epileptic encephalopathy, early infantile, 4	612164	1934	1/100000
	STXBP2	Hemophagocytic lymphohistiocytosis, familial, 5	613101	540	1/100000
	STXBP3				
sec2	RAB3IP				
	RAB3IL1				
sec3	EXOC1				
sec4	RAB8A				
	RAB8B				
sec5	EXOC2				
sec6	EXOC3				
sec7	ARFGEF1				
	ARFGEF2	Periventricular heterotopia with microcephaly	608097	98892	
sec8	EXOC4				
sec9	SNAP25	Myasthenic syndrome, congenital, 18	616330	590	
	SNAP29	Cerebral dysgenesis, neuropathy, ichthyosis, and palmoplantar keratoderma syndrome	609528	66631	7 subjects identified
	SNAP23				
sec10	EXOC5				
sec11	SEC11C				
	SEC11A				
	SEC11B				
sec12	PREB				
sec13	SEC13				
sec14	SEC14L2				
	SEC14L3				
	SEC14L1				
	SEC14L5				
	SEC14L4				
	SEC14L6				
sec15	EXOC6B	Spondyloepimetaphyseal dysplasia with joint laxity, type III	618395	93359	
	EXOC6				
sec16	SEC16A				
	SEC16B				
sec17	NAPA				
	NAPB				
sec18	NSF				
sec20	BNIP1				
sec21	COPG1				
	COPG2				
sec22	SEC22B				
	SEC22C				
	SEC22A				
sec23	SEC23A	Craniolenticulosutural dysplasia	607812	50814	27 cases described
	SEC23B	Cowden syndrome 7	616858	201	1 in 200,000 to 250,000
	SEC23B	Dyserythropoietic anemia, congenital, type II	224100	98873	

An alternate view is that the study of natural rare mutations in humans may be a risky choice. Arguments in favor of this view center around three chief arguments. First is the fact that humans are not genetically homogeneous. However, genomic efforts to understand the impact of mouse genetic diversity on phenotypic outcomes will help us to assess the impact of the genetic heterogeneity inherent to the study of human genetic diseases (Srivastava et al., 2017). Second, human mutations can be exceedingly rare, thus preventing a large casuistic for study. Third, some human mutation-associated phenotypes are not systematically annotated or quantitative. Despite these caveats, rare mutations in human models represent an opportunity much like the *cdc*, *sec*, or *period* genes and their mutations. Rare human genetic diseases can be seen as the results of the forward genetic screen that nature has been continuously running on us since the emergence of our species. These mutations and their phenotypes tell us incontrovertibly that these genes matter for a process yet to be discovered. The risk versus reward dilemma in the study of rare human diseases is articulated elegantly by Carl Zimmer in an article describing the rare disease fibrodysplasia ossificans progressiva (Zimmer, 2013). The prevalence of this disease is 1 in 2 million, a fact that could easily become a deterrent for study. Yet the penetrant and severe phenotype and therefore the mechanisms underneath the phenotype were hard to ignore for the pioneers studying this ultra-rare disease. Fibrodysplasia ossificans progressiva results in heterotopic ossification of muscle and connective tissue, a phenotype caused by dominant mutations in the gene ACVR1, encoding the activin A receptor 1 (OMIM 135100). ACVR1 is a widely expressed and evolutionarily recent gene appearing in mammals. The ACVR1 activity is required for controlling growth and development of bones and muscles, including endochondral ossification (Kaplan et al., 2012). The ACVR1 disease-causing mutations increase signal transduction through the bone morphogenetic protein signal transduction pathway whose ligands bind to ACVR1. This biological insight offered by the discovery of ACVR1 mutations in humans precedes information obtained from model genetic organisms (Kaplan et al., 2012, Shore et al., 2006).

Thus, if a mutation causes a strong and penetrant phenotype, the low prevalence of a rare genetic mutation in model genetic organisms or humans should not be a deterrent for their consideration. The frequency of a genetic defect neither foresees the significance of the biological process gone awry nor does it predict the potential for impacting human biology and health. We will discuss this point later using Menkes disease as an example. The pursuit for understanding human mutations with robust and penetrant phenotypes is a demonstrated path for unraveling universal biological principles much like forward genetic screens performed in isogeneic genetic organisms. This idea is not new. It was first formulated by William Harvey in 1657 in a response to John Vlackveld of Harlem, a Dutch physician who was asking Harvey's advice concerning a unique clinical case:

Harvey replied: *“It is even so-Nature is nowhere accustomed more openly to display her secret mysteries than in cases where she shows traces of her workings apart from the beaten path; nor is there any better way to advance the proper practice of medicine than to give our minds to the discovery of the usual law of Nature by careful investigation of cases of rarer forms of disease. For it has been found, in almost all things, that what they contain of useful or applicable is hardly perceived unless we are deprived of them, or they become deranged in some way.”*

Harvey's vision was first enunciated in the context of genetic diseases by Archibald Garrod, the father of Medical Genetics in 1928 (Garrod, 1928). Garrod also paraphrased this concept stating:

“The study of nature's experiments is of special value; and many lessons which rare maladies can teach could hardly be learned in other ways.”

In the next sections, we attempt to provide some answers to these questions posed then and now: What lessons can be learned from diseases as individual entities, and what can we learn from their collective study?

## Learning from Rare Genetic Diseases through Systems Biology

It is estimated that 6,172 clinically distinct diseases are genetic in nature according to Orphanet, an online database of rare diseases that includes genetic and non-genetic diseases defined according to the European prevalence threshold (Cutillo et al., 2017). As of early 2020, the Online Mendelian Inheritance in Man resource, OMIM, lists 6,594 diseases with known molecular genetic defect, which collectively encompass 4,225 genes (https://www.omim.org/statistics/geneMap). This figure is increasing at a rate of ∼50–60 new genetic diseases per year in the Orphanet and OMIM databases (Boycott et al., 2017). Initiatives such as the NIH Undiagnosed Diseases Program and Network and The International Rare Diseases Research Consortium are accelerating the rate of discovery of novel genetic diseases (Gahl et al., 2016, Kuehn, 2011). Thus, it is conceivable that with time, we may identify sufficient genetic defects in humans so we can come close to the desired goal of all geneticists, a saturation mutagenesis screen to hit all genes and to find phenotypes of every individual gene.

**Figure 2:**
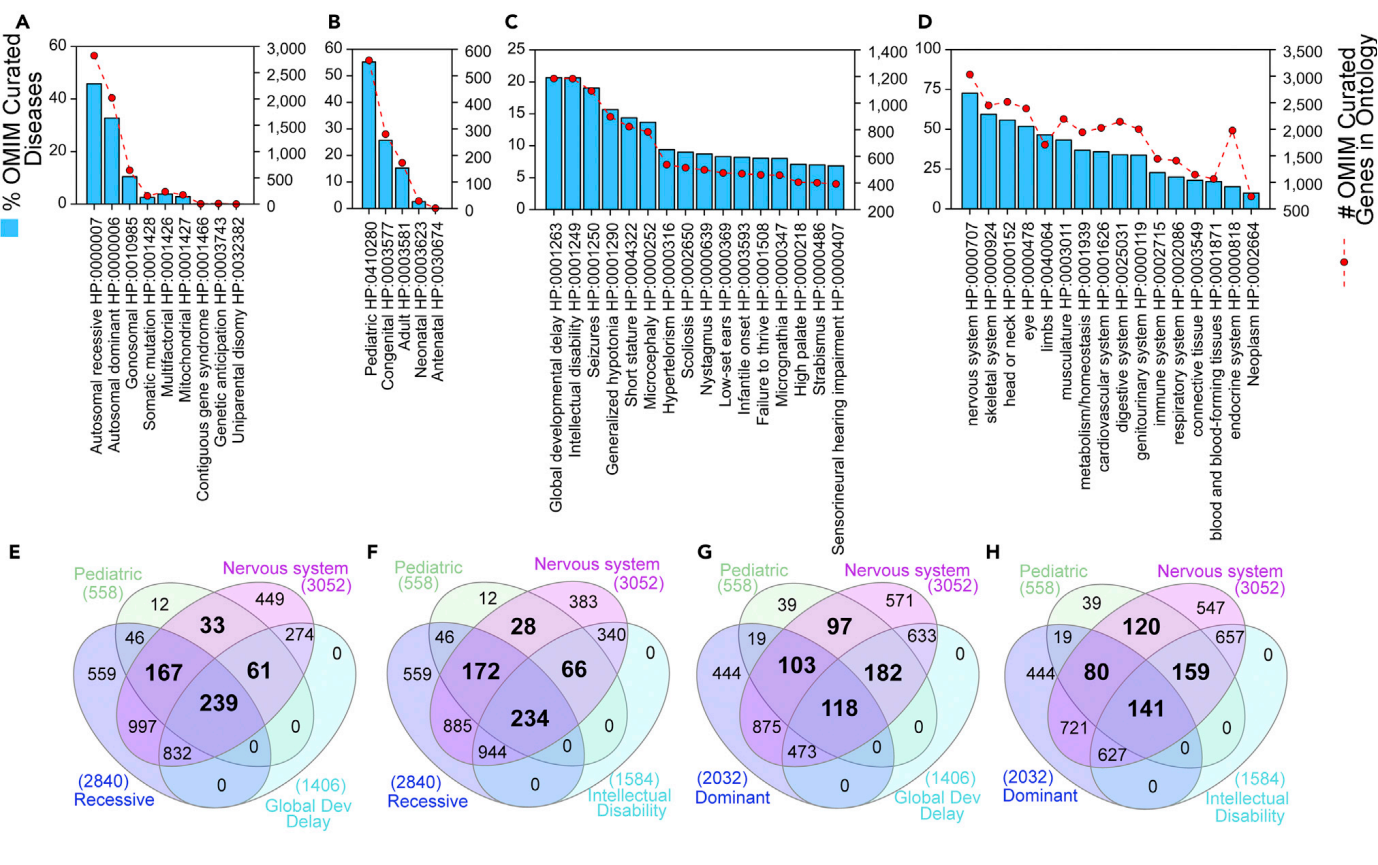
Quantitative Descriptors of Rare Genetic Disorders Curated by OMIM and Annotated by HPO We used the HPO annotated descriptors for all curated genetic disorders to assess global descriptors of disease. (A) Describes all diseases and associated genes according to Mode of inheritance HP:0000005. (B) Presents the distribution of diseases according to the age group in which disease manifestations appear: Onset HP:0003674. (C) Diseases were classified according to annotated clinical phenotypes. (D) Graph presents the distribution of disease according to the organ/tissue/system affected. Phenotypic abnormality HP:0000118. (E–H) Venn diagrams present overlaps between different HPO terms listed in A–D. All bold numbers indicate overlaps of diseases with HPO terms associated to childhood converging on nervous system and behavioral HPO terms. (E and F) present data for recessive disorders. (G and H) depict data for dominant disorders. (A-D) Y axis represent % of curated OMIM diseases and Y1 axis shows the number of genes associated to the HPO terms (red symbols).

To extract information out of these ∼6,000 rare genetic diseases, we analyzed genetic diseases listed in OMIM using disease descriptors from the Human Phenotype Ontology Database (HPO). The March 2020 release of the HPO offered us ∼156,000 annotations to rare diseases using a palette of over 13,000 ontological descriptors (Kohler et al., 2017). Of the 6,594 OMIM disease entries, 45% correspond to autosomal recessive diseases (HP:0000007), 32% fall into autosomal dominant disease category (HP:0000006), and 10% are diseases linked to the X and Y chromosomes (HP:0010985) (Figure 2A).

Analysis of the age of onset in rare diseases (HP:0003674) reveals the remarkable observation that 55% of all diseases are of pediatric origin (HP:0410280, Figure 2B), an onset category defined as diseases that manifest before the age of 16 years, but excluding neonatal or congenital onset. However, if we pool together pediatric, congenital, and neonatal diseases (referred here as childhood diseases); a staggering 83% of all rare diseases affect the child (Figure 2B). The picture is similar if we consider other ontological terms that capture diverse phenotypic manifestations in all rare diseases. The two top ontological terms encompassing 40% of all rare genetic diseases are by definition ascribed to children. These include global developmental delay and intellectual disability (HP:0001263 and HP:0001249, Figures 2E–2H), which describe delays in achieving motor or mental milestones before puberty. Similarly, analysis of all rare genetic diseases by the organ these diseases affect indicate that, irrespective of age, close to 70% of these diseases produce abnormalities of the nervous system (HP:0000707, Figure 2D). Our findings are in rapport with a recently reported study (Sanders et al., 2019). The compromise of the nervous system is even more pronounced in childhood diseases where 89.6% of childhood diseases compromise the nervous system or behavior irrespective of whether these diseases are dominant or recessive (Figures 2E–2H, bold numbers). These findings show that rare diseases preferentially affect human development and in particular the development of the nervous system.

## Unifying Principles of Rare Genetic Diseases, a Systems Biology Perspective

**Figure 3:**
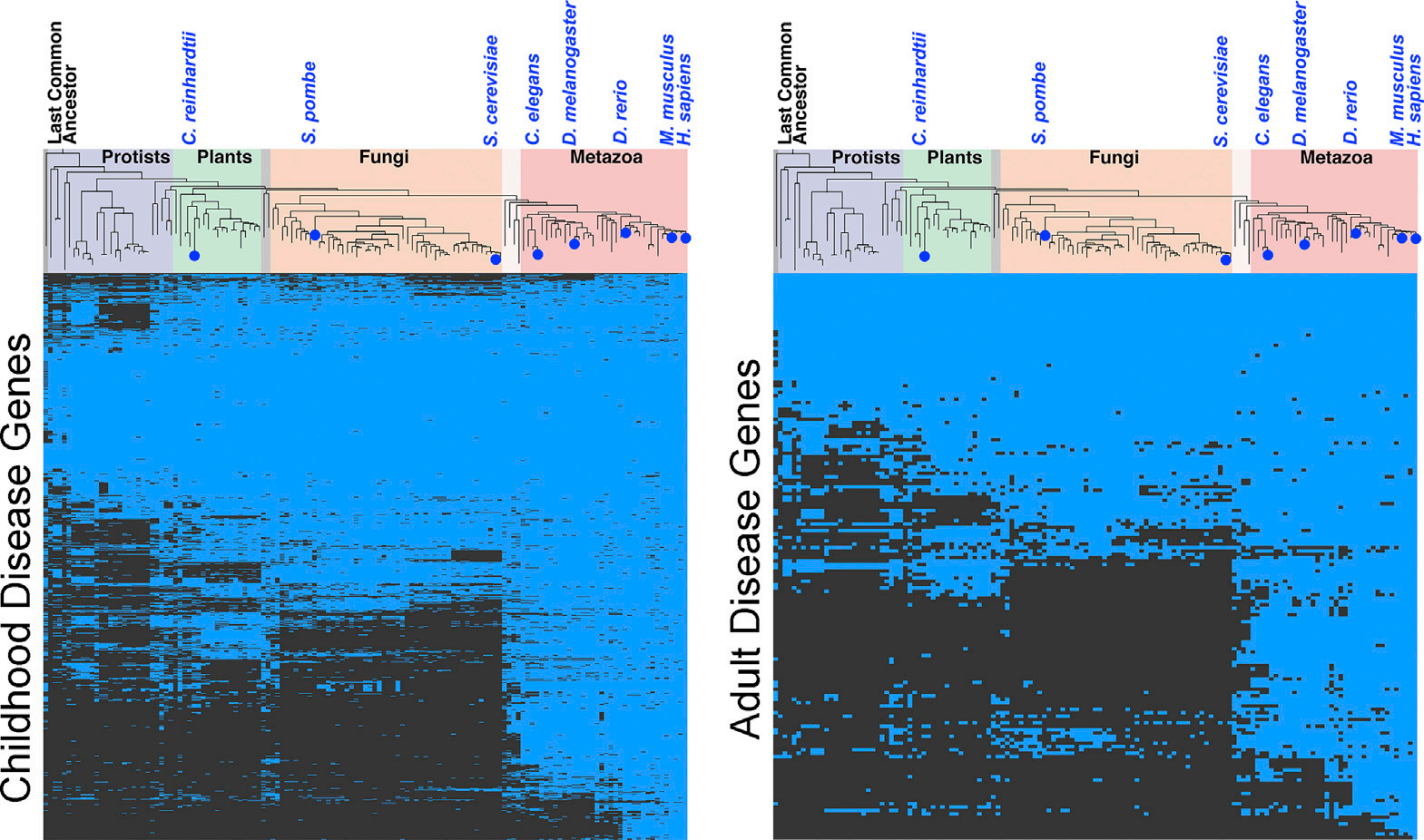
Most Rare Disease Genes are Evolutionarily Ancient We use the lists of genes associated with childhood diseases (pooled HP:0410280, HP:0030674, HP:0003577, and HP:0003623) and adult genetic diseases (HP:0003581) and analyzed them with the CLIME engine to determine the presence of orthologues and paralogues across species (Li et al., 2014). The presence of paralogues or orthologues is marked by blue cells in the heatmap.

The above findings beg the question of why the most prevalent phenotypes among rare diseases are abnormalities of the nervous system that disproportionally affect the child. We used systems biology analyses of genes affected in diseases of the childhood and compared them with genes associated to diseases of the adult. We chose childhood and adult categories because these disease onset descriptors are annotated in the HPO database (see legend to Figure 3 and HP:0003674). We discriminated among the following hypotheses that could account for this phenotypic bias toward the child nervous system:

1) Genes associated with childhood diseases appeared in evolution together with the emergence of nervous systems in metazoans.
2) Childhood disease genes are preferentially enriched in the developing brain.
3) Childhood disease genes are preferentially expressed in neurons or glia and their subcellular compartments, such as the synapse.
4) Childhood disease genes code for proteins that form nodes of high interconnectivity in protein interaction networks.

This latter idea is founded on the observation that highly connected nodes tend to enrich gene products in which mutations produce lethality or impair housekeeping functions, suggesting that these genes are physiologically indispensable (Jeong et al., 2001, Lin et al., 2009, Rodrigues and Costa Lda, 2009, Yang et al., 2014, Yang et al., 2016).

**Figure 4:**
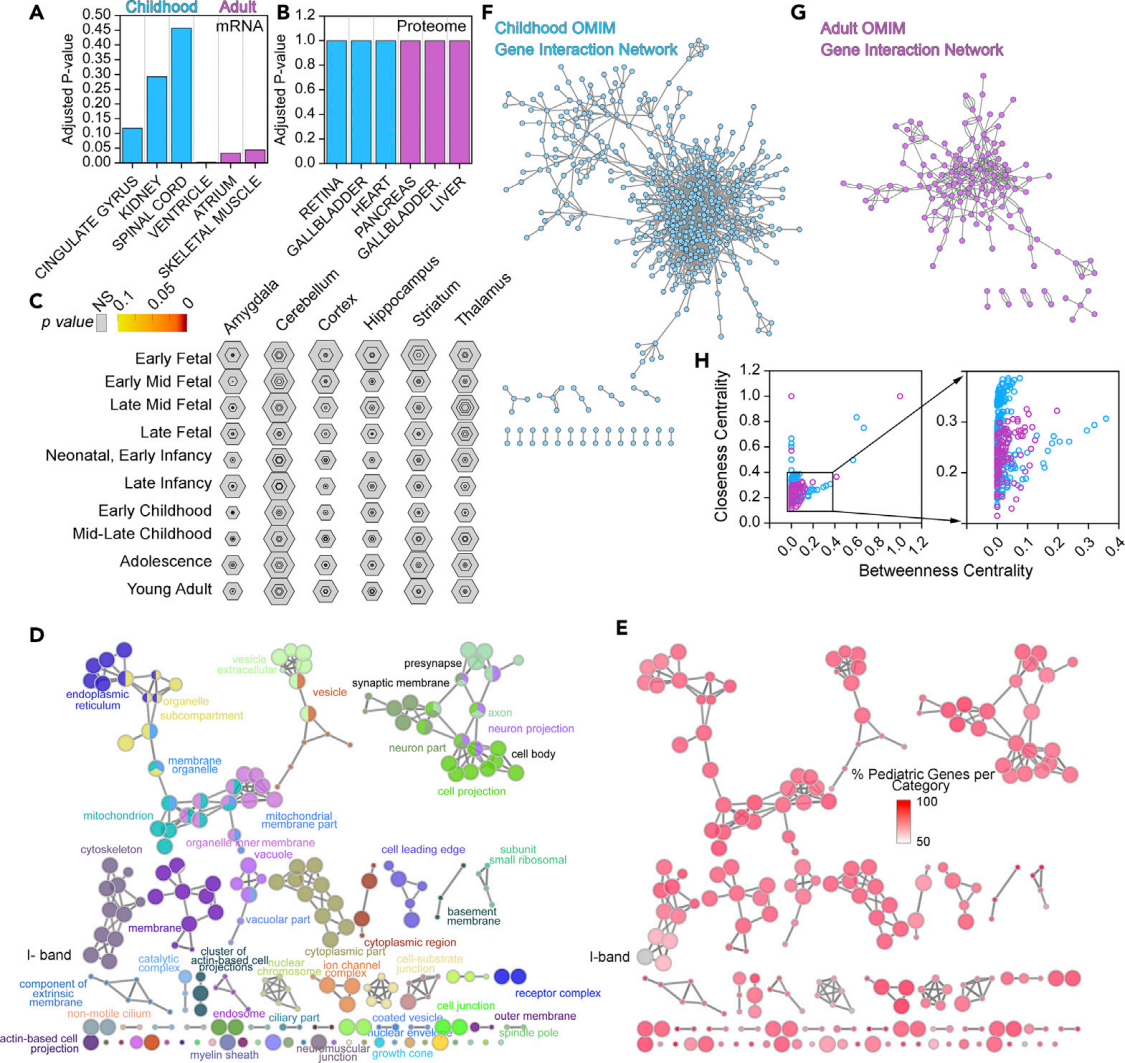
Rare Disease Gene Ontologies and Protein-Protein Interaction Topologies (A–C) We used the childhood and adult gene HPO lists to explore tissue expression in human tissues using the ARCHS4 transcript database (A), the Human Proteome Map Database (B), or de human brain developmental mRNA expression database CSEA (C) (Kim et al., 2014, Lachmann et al., 2018, Wells et al., 2015). A and B present top ranked tissues where gene lists are expressed. Note that only adult disease mRNAs are significantly enriched in categories describing striated muscle. Childhood diseases genes do not enrich nervous tissue or any other tissue ontologies. Figures A–C show Fisher's exact p values, followed by the Benjamini-Hochberg correction. (D and E) Gene lists for childhood and adult diseases were combined and analyzed using the Cytoscape ClueGo plugin for cellular compartment ontologies (GO:CC) (Bindea et al., 2009). Color in D depicts GO CC terms. Color in E represents the percentage of genes that belong to childhood diseases in the CC term depicted in (D). Note that only one term is equally represented by childhood and adult genes: the I- band belonging to striated muscle. All ontologies are significant with a corrected p value <0.05. Size of circle is proportional to the significance of the term. (F–H) Protein-protein interaction network data for the childhood (F) and adult disease gene lists (G) were obtained from Genemania (Franz et al., 2018). Networks were built and their topologies analyzed with Cytoscape and the NetworkAnalyzer plugin (Doncheva et al., 2012, Shannon et al., 2003). H presents centrality parameters for the childhood (blue symbols) and adult disease genes (purple symbols).

Are rare diseases genes associated with childhood or adult genetic diseases a recent or ancient evolutionary occurrence? We used the CLIME engine to analyze the evolution of childhood or adult disease genes (Li et al., 2014)(Figure 3). Sixty percent of all childhood and adult genes possess orthologues in organisms that lack nervous systems including unicellular plants (*Chlamydomonas reinhardtii*) or unicellular fungi (*Saccharomyces cerevisiae*). However, just 5% of these childhood and adult genes appeared together with the emergence of metazoans with complex nervous systems, such as *Drosophila melanogaster*. This enrichment in ancestral genes among rare genetic disorders likely explains the exceedingly low prevalence of these diseases due to early embryonic lethality. In fact, it is estimated that between 50% and 70% of all early miscarriages are associated with aneuploidies and with 66 monogenic defects annotated in the HPO database (HP:0005268) (Hyde and Schust, 2015, Soler et al., 2017, van den Berg et al., 2012). These miscarriage-associated genes are enriched in constituents of the axoneme, an ancient organelle present since the emergence of unicellular plants (GO:0005858, p= 7.603 × 10^−12^)(Carvalho-Santos et al., 2011). In agreement with these evolutionary findings, we observed that the expression of childhood rare disease genes is not significantly enriched in the brain or any brain regions across development (Figures 4A–4C). Moreover, organelle-based ontologies could not distinguish childhood and adult diseases (Figure 4D). In fact, there is a balanced representation of organelles present in all cell types such as mitochondria, peroxisome, or vacuole. These ubiquitous organelles are similarly represented as compared with neuronal subcompartments (Figure 4D). Childhood diseases genes were overrepresented in all these cellular compartment annotations as compared with genes contributed by adult diseases (Figure 4E). The only exception was the cellular compartment, I-band, a muscle sarcomere structure (GO:0031674, Figure 4D, gray color), where both childhood and adult disease genes were annotated in a 1 to 1 ratio. Genes from childhood and adult diseases were annotated to neuronal compartment terms, such as synapse, in a 4 to 1 ratio (GO:0045202). However, the rare disease gene ratio is even more pronounced with a 1 to 0 and 5.4 to 1 ratio between childhood and adult disease genes for genes that are annotated to the peroxisome (GO:0044439) or mitochondria, respectively (GO:0005739). Genetic defects in these last two ubiquitous cellular organelles have long been recognized as severely affecting the nervous system of the child even though these organelles are present in all eukaryotes and metazoan tissues (Gorman et al., 2016, Koumandou et al., 2013, Waterham et al., 2016). These analyses allow us to draw two general conclusions. First, genes necessary for fundamental cellular processes are likely required throughout the lifespan of the organism, thus providing an explanation to the early life appearance of rare genetic disease phenotypes. Second, our evolutionary, tissue, or subcellular compartment criteria neither explain the preponderance of neurological and behavioral phenotypes in all genetic diseases combined nor in genetic diseases of the childhood.

In seeking answers to the disproportionate effects of rare genetic disease on the nervous system among diseases of the childhood (Sanders et al., 2019), we asked if protein-protein interaction networks constructed with childhood gene products differed in complexity from networks assembled with genes causative of rare diseases of the adult. We used experimentally defined protein-protein interaction networks and integrated them with predicted protein-protein interaction networks generated by yeast two-hybrid analyses. These interaction networks have been curated, maintained, and updated in the Genemania web engine (Franz et al., 2018) (Figures 4F and 4G). The network generated with childhood disease genes has higher network heterogeneity index than the adult network (1.476 versus 0.818), indicating that the childhood disease gene network contains more “hub” protein nodes than the adult network. Furthermore, the childhood disease gene network possesses protein nodes of a higher connectivity to other nodes within the network, as revealed by the average number of neighbors, 4.5 neighbors per node in the childhood disease network versus 3.4 for adult disease network proteins nodes. These global network parameters indicate that the childhood disease protein-protein interaction network is more complex and its nodes are of higher connectivity than those in the adult network.

We further determined the extent of childhood and adult network complexity asking how each protein connected to the rest of protein nodes using centrality parameters (Figure 4H). We described each protein node with betweenness and closeness centrality scores. Betweenness centrality of a protein node quantifies the amount of control that this node exerts over the interactions of other nodes within the network. Closeness centrality is a measure of how close a node is to other nodes, an indication of how quickly information spreads from a given node to other reachable nodes in the network (Doncheva et al., 2012, Dong and Horvath, 2007, Freeman, 1979, Yoon et al., 2006). Childhood disease protein nodes displayed higher centrality indexes as compared with adult disease protein nodes. This demonstrates that childhood disease proteins are biased toward more complex interactions than adult disease proteins. This begs the question, how are the complexity of the childhood disease networks and the preponderance of neurological phenotypes related to each other? One view is that genes expressed in the brain are engaged in networks as nodes of higher connectivity. However, present data argue against this hypothesis, as brain protein-protein interactions are comparable in connectivity to other tissues (Barshir et al., 2014). Rather it seems that widely expressed proteins mutated in genetic diseases are more likely to engage with other proteins involved in tissue-specific protein-protein interactions (Barshir et al., 2014). This observation is in line with recent findings demonstrating that tissue-specific proteins bridge conserved protein complexes present in most tissues (Bossi and Lehner, 2009, Huttlin et al., 2020, Luck et al., 2019). The brain is the most diversified proteome of all organs and the human organ with the second most tissue-enriched/specifically expressed proteins (Fagerberg et al., 2014, Sharma et al., 2015, Sjostedt et al., 2015, Uhlen et al., 2015). Thus, we propose that the prevalence of childhood diseases affecting nervous tissue is the product of the topological complexity of the networks formed by ubiquitous proteins encoded by childhood disease genes plus the abundance of brain-enriched/specific protein. We speculate that brain-enriched/specific proteins would propagate the consequences of genetic defects in a conserved protein complex into other conserved and ubiquitous complexes, thus amplifying the emergence of phenotypes in the brain.

We conclude that investigating rare genetic diseases is in fact a way to study the function of the most evolutionarily conserved genes and cellular mechanisms. Once again, similar to the case with Harvey and Garrod illustrate, this is not a new idea. It has long been recognized that ascertainment of monogenic genetic diseases by rare and early onset cases is a tool to study common yet genetically complex diseases. This is the case of Alzheimer, schizophrenia, or type II diabetes where pedigrees of families with early age onset of disease have been instructive of the mechanisms of common complex diseases that are frequently polygenic and influenced by environmental factors (Fajans et al., 2001, Murrell et al., 1991, Rapoport et al., 2012).

## Learning about Common Human Diseases from a Rare Genetic Disease

Menkes disease exemplifies central features of most rare genetic diseases. Menkes is a childhood multisystemic disease that severely affects the nervous system (OMIM 309400). As other examples discussed earlier, the study of Menkes disease has opened the door to fundamental concepts about the molecular biology and physiology of trace metals. Even after 58 years of studying this disease, there are still Menkes-inspired novel biological insights. Recently, we produced evidence of genetic and molecular interactions between the Menkes disease gene, ATP7A, and common diseases such as Parkinson disease (Comstra et al., 2017, Hartwig et al., 2019, Zlatic et al., 2018).

The story of Menkes disease began in New York in 1962 with a family of English-Irish descent. Dr John H. Menkes described the first five patients belonging to this family. His patients were affected by an X-linked recessive disease characterized by failure to thrive, abnormal hair and skin, intellectual disability, as well as cerebral and cerebellar neurodegeneration. Severe neurologic symptoms appeared 1–2 months after birth and progressed rapidly to death (Menkes, 1988, Menkes et al., 1962). The next chapter began in 1972, when David Danks et al. demonstrated low levels of serum copper and ceruloplasmin in seven patients with Australian Menkes disease, leading to Danks' seminal proposition that Menkes disease is a disease of copper absorption (Danks et al., 1972). This hypothesis was definitively tested with an enterocyte-specific knock-out of the murine *Atp7a* gene that fully recapitulates Menkes disease (Wang et al., 2012). Notably, human-research-founded hypotheses preceded the identification of the metabolic defect in mice that carry *Atp7a* mutations by decades with the study of the mottled series of mice (Grimes et al., 1997, Hunt, 1974). It took 21 years after the disease's first description for three groups to independently clone the candidate gene of Menkes disease, ATP7A, a finding that spurred our understanding of copper biology (Chelly et al., 1993, Mercer et al., 1993, Vulpe et al., 1993). Even before the identification of the gene mutated in Menkes disease, Danks presciently concluded that the study of this rare disease would lead to “*many new lines of research on copper metabolism and trace metal deficiency.*” This statement abides by the spirit of Harvey and Garrod dictum about the conceptual gains of studying Nature's defects that fall "*apart from the beaten path"* (Danks et al., 1972).

Presently, we know that genetic defects affecting the copper-transporter P-ATPase, ATP7A, cause three X-linked recessive rare diseases: occipital horn syndrome (OMIM 304150), spinal muscular atrophy, distal, X-linked 3 (SMAX3, OMIM 300489), and Menkes disease (OMIM 309400) (Kaler, 2011). Approximately 380 different mutations affecting ATP7A gene have been described so far in the Human Gene Mutation Database (Moller et al., 2009, Tumer, 2013). Milder missense mutations in ATP7A generate occipital horn syndrome, which is characterized by ataxia, dysarthria, moderate hypotonia, and intellectual disability in addition to systemic phenotypes (Das et al., 1995, Kaler et al., 1994), and SMAX3, where spinomuscular atrophy occurs independently of cognitive defects (Kennerson et al., 2010, Takata et al., 2004). More severe loss-of-function mutations result in Menkes disease proper, a multisystemic metabolic disease affecting copper homeostasis. Menkes is a rare affliction with an incidence of 1/140,000 to 1/300,000 (Gu et al., 2005, Kaler, 2011). Menkes manifests soon after birth with hypotonia, focal and generalized seizures, impaired cognitive development, and brain atrophy. Systemically, Menkes disease affects hair with multiple defects including *pili torti* (twisted hairs), monilethrix (beaded hairs), and thickened or weak nodes that cause hair fragility (trichorrhexis nodosa). In addition, hair and skin are hypopigmented. There is laxity of the skin (*cutis laxa*) and joints, osteoporosis, bladder diverticula, aneurysms, and vascular tortuosity in brain arteries (Kaler, 2011, Lutsenko et al., 2007, Manara et al., 2017, Polishchuk and Lutsenko, 2013).

Menkes disease clinical features have been traditionally attributed to defects in diverse cuproenzymes that traverse the secretory pathway and remain as inactive apoenzymes in the disease state (Kaler, 2011, Lutsenko et al., 2007, Polishchuk and Lutsenko, 2013) (Table 2). This defect in loading copper into apoenzymes results from a defect in copper transport into the Golgi lumen as ATP7A normally is a resident transporter of the Golgi apparatus (Petris et al., 1996). The cutis laxa, bone, bladder, and vascular phenotypes are attributed to the defective activity of enzymes required for the modification of collagen fibers and elastin such as lysyl oxidase (LOX). Hypopigmentation is due to defective tyrosinase activity and hair defects to impaired sulfhydryl oxidase activity (Harris, 2013, Tumer and Moller, 2010, Zlatic et al., 2015). The success of this enzymatic model explaining systemic phenotypes in Menkes disease has been extended to the neurological symptoms in Menkes disease (Zlatic et al., 2015). It has been proposed that defective enzymatic activities of cytochrome *c* oxidase, dopamine β-monooxygenase, and peptidyl-α−amidating monooxygenase are responsible for the nervous system defects in Menkes disease (Menkes, 1988, Zlatic et al., 2015). These enzymes play major roles in mitochondrial respiration, neurotransmitter, and neuropeptide biosynthesis respectively. We termed this enzymatic model of neurological phenotypes the oligoenzymatic hypothesis, which we consider insufficient to explain Menkes neuropathology (Kaler, 2011, Menkes, 1999, Zlatic et al., 2015). In fact, the interactome of the Menkes ATPase ATP7A and the proteome of ATP7A null cells are enriched in gene products involved in neurodegenerative and neurodevelopmental diseases, suggesting a larger complexity to the pathogenesis mechanisms in Menkes neurological disease (Comstra et al., 2017, Hartwig et al., 2019, Zlatic et al., 2018).

**Table 2:** Copper-Dependent Enzymes and Phenotypes Associated in Menkes Disease

Enzyme	Gene	Compartment	Biological Activity	Symptom
Cytochrome c oxidase	Varios Genes	Mitochondria	Cellular respiration	CNS degeneration?
	See CORUM Complex #6442			Ataxia?
		
		
Superoxide dismutase	SOD1	Mitochondria	Free radical scavenging	CNS degeneration?
Tyrosinase	TYR	Lysosome-related organelles	Pigment formation	Hypopigmentation
Dopamine β-hydroxylase	DBH	Large dense core secretory vesicles	Catecholamine production	Ataxia?
				Hypothermia
				Hypotension
		
Peptidylglycine alpha-amidating monooxygenase	PAM	Large dense core secretory vesicles	Activation of peptide hormones	Wide spread effects, vascular and pancreas defects
Lysyl oxidase	LOX	Secreted	Collagen and elastin cross-linking	Premature rapture of fetal membranes
				Cephalohematoma
				Abnormal facies
				High-arched palate
				Emphysema
				Hernias
				Bladder diverticula
				Arterial aneurysms
				Loose skin and joints
				Osteoporosis
				Petechial hemorrhage
				Poor wound healing
				CNS degeneration?
Sulfhydryl oxidase	Possibly QSOX1 and 2	Golgi and secreted	Cross-linking of keratin	Abnormal hair
				Dry skin

Adapted from (Harris, 2013, Tumer and Moller, 2010, Zlatic et al., 2015).

Despite the fact that Menkes disease is a rare genetic disease, we believe Menkes continues to be a tool to uncover novel and fundamental knowledge regarding mechanisms underlying metal-dependent neurodegeneration in common diseases. Copper imbalances exacerbate the magnitude and progression of neurodegenerative diseases (Davies et al., 2014, Davies et al., 2016, Dusek et al., 2015, Lorincz, 2010), with even minimal exposure to copper at dietary levels sufficient to trigger neuropathology and cognitive decline (Sparks and Schreurs, 2003). Menkes is also a model to identify mechanisms shared with neurodegenerative and neurodevelopmental diseases where environmental exposures, such as redox active metals, are powerful risk factors. For example, copper content alterations are often found in neurodegenerative diseases such as in Parkinson and Parkinsonism (Davies et al., 2014, Davies et al., 2016, Dusek et al., 2015, Genoud et al., 2019, Lorincz, 2010). Thus, as is the case in other rare diseases, the study of Menkes disease has the potential to intersect with and reveal new mechanisms associated with prevalent diseases.

## Conclusions

The lessons that rare genetic diseases teach us continue to affirm the dictums of Harvey and Garrod: We can do “*discovery of the usual law of Nature by careful investigation of cases of rarer forms of disease*”. Rare genetic diseases disproportionately affect the nervous system of children with devastating effects. Paradoxically, the majority of the disease-causing genes affecting the child belong to genes present from the last common eukaryote and ubiquitously expressed in human tissues. Thus, whether we study a mutation in a yeast, fly, or a human, we argue we are in essence studying the same principles across the radiating richness given to us by evolution.

The concept of rare diseases focuses on a seemingly low percentage of people affected by a single affliction. Collectively, however, these *rare* diseases affect nearly 4% of the people during their lifetime. We predict that the numbers of patients with rare disease will dramatically increase in the new era of the truly rare disease: the disease of one-and-only-one individual. This concept is intrinsic to the idea of personalized medicine (Collins and Varmus, 2015). If personalized medicine delivers on its promises, the future of medicine is bound for the study and treatment of the rarest of all diseases, the disease of the “I” rather than the “us.” The principles that we have articulated for rare diseases here will be equally applicable for personalized medicine. The richest horizon for biological exploration may be just around the corner, where the information of every human genome will be tied to the individual's normal or pathological traits.
